# Psychological well‐being and the reversal of childhood overweight and obesity in the UK: a longitudinal national cohort study

**DOI:** 10.1002/oby.24147

**Published:** 2024-10-07

**Authors:** I Gusti Ngurah Edi Putra, Michael Daly, Eric Robinson

**Affiliations:** ^1^ Department of Psychology, Institute of Population Health University of Liverpool Liverpool UK; ^2^ Department of Psychology Maynooth University Kildare Ireland

## Abstract

**Objective:**

This study aimed to examine the prospective association between psychological well‐being and overweight and obesity reversal.

**Methods:**

We analyzed data of UK children with overweight or obesity at ages 11 (*n* = 4556, baseline), 14 (*n* = 3791, baseline), and 17 years (follow‐up). Psychological well‐being‐related measures were characterized into indexes of caregiver‐reported child mental health and child‐reported psychosocial well‐being, with a higher score indicating better mental health or psychosocial well‐being. Weight changes were presented as reversal versus persistence of overweight or obesity and residualized‐change BMI *z* scores. Data were analyzed using regression analysis.

**Results:**

Better child mental health and psychosocial well‐being at age 11 years were independently associated with increased odds of reversal versus persistence (odds ratio [OR] = 1.16, 95% CI: 1.03 to 1.29; OR = 1.29, 95% CI: 1.15 to 1.44, respectively) and decreased BMI *z* scores (β = −0.08, 95% CI: −0.13 to −0.03; β = −0.07, 95% CI: −0.11 to −0.03, respectively) at age 17 years. However, neither of the indexes was associated with weight changes when measured at age 14 years. Analyses between psychological well‐being‐related measures and timing of measures indicated that psychological well‐being‐related measures were more likely to prospectively predict weight changes when measured at age 11 versus age 14 years.

**Conclusions:**

Better psychological well‐being at age 11 years is a prognostic factor that may be associated with an increased likelihood of reversing childhood overweight and obesity by age 17 years.

**Study Importance**

**What is already known?**

- Poor psychological well‐being is associated with increased risk of developing overweight and obesity.
- There is a dearth of evidence on whether better psychological well‐being is associated with overweight and obesity reversal.

**What does this study add?**

- Better child mental health and psychosocial well‐being at age 11 years, but not age 14 years, are associated with overweight and obesity reversal at age 17 years.
- Age 11 years may be a sensitive period in which psychological well‐being is associated with future body weight trajectories.

**How might these results change the direction of research or the focus of clinical practice?**

- Our findings inform the importance of exploring the role of psychological well‐being in predicting the reversal of overweight and obesity and understanding potential mechanisms.
- Integrating psychological support into current obesity prevention or weight loss strategies, particularly during an age‐sensitive period, may be important.

## INTRODUCTION

Childhood obesity is an important public health challenge because it tracks into adulthood and is associated with future detrimental health outcomes, including cardiovascular diseases and increased mortality risk [1, 2]. Extensive research has shown that impaired psychological well‐being plays a significant role in the development of overweight and obesity [3]. However, it is not yet clear whether a negative state of psychological well‐being that encompasses a range of constructs related to individuals' feelings or emotions (e.g., self‐esteem, happiness, depressive symptoms) and functioning in personal and social aspects (e.g., positive relationships, mastery) [4, 5] is also responsible for the persistence of overweight and obesity once developed in childhood.

In adults, overweight or obesity reversal is thought to be relatively rare [6]. Sustained biological changes due to weight gain, such as cellular leptin resistance and reduced postprandial suppression of ghrelin, may explain why weight loss is difficult [7, 8, 9]. In addition, chronic changes in gut hormones associated with increased appetite and reward value of food may maintain overweight and obesity during adulthood [9]. There is significant tracking of weight status across childhood to young adulthood, whereby children with obesity are around five times more likely to have obesity during adulthood [1]. However, it is less well recognized that overweight or obesity is reversed in a significant proportion of children by the end of adolescence. For example, in a UK study, more than 20% of children with overweight or obesity at age 11 years were classified as having normal weight by age 14 years [10].

To date, there is a lack of research seeking to understand why some children experience reversal versus persistence of overweight and obesity. Psychological well‐being may play an important role. Mental health difficulties and negative psychosocial outcomes such as depressive symptoms and low self‐esteem are more prevalent among children living with overweight and obesity [11]. Behavioral responses to these mental health difficulties and negative psychosocial outcomes, such as unhealthy eating and reduced physical activity [12, 13, 14, 15, 16], may then contribute to persistence of overweight and obesity. In addition, exposure to negative psychosocial stressors (e.g., weight stigma) in children with overweight or obesity may increase cortisol secretion [17], which causes fat accumulation [18]. For these reasons, better mental health and psychosocial well‐being may be important factors that enhance the likelihood of reversal of overweight and obesity in children. In line with this, previous studies in adults have identified psychological well‐being‐related measures (e.g., body image, depression) as being predictive of weight loss [19]. However, there is a dearth of evidence on this in children.

A previous study in UK children examined the role of early puberty and exercise at age 11 years in overweight and obesity reversal at age 14 years [10], but the role of psychological well‐being‐related measures was not investigated. Other studies have found that self‐esteem and behavioral problems [20, 21] were associated with changes in body mass index (BMI) *z* scores in children. However, no studies, to our knowledge, have examined whether a wide range of psychological well‐being‐related measures is associated with persistence of reversal of overweight or obesity during childhood. This will be important for informing current obesity prevention policies and weight loss interventions in children and adolescents. To address this gap, we used nationally representative cohort data of UK children, i.e., the Millenium Cohort Study (MCS), to investigate the role of a broad range of psychological well‐being‐related measures in predicting reversal versus persistence of childhood overweight and obesity.

MCS includes psychological well‐being‐related measures, which assess both mental health and what we describe as “psychosocial well‐being.” Mental health in this study refers to a mental state including healthy behavioral adjustment, emotional well‐being, and minimal disabling symptoms and anxiety [22]. Psychosocial well‐being encompasses social, cultural, psychological, and subjective components that together influence overall well‐being, including how individuals function and reach their full potential within society [23, 24]. Although “mental health” and “psychosocial well‐being” terms overlap in some contexts [24], we used these terms in the present work to explore their distinct predictive contributions. Recent research has suggested that comorbidity between body weight and mental health problems (e.g., internalizing symptoms) is minimal during early childhood (i.e., ages <7 years) [25]. Therefore, in the present study, we capitalize on all available MCS data collected from ages 11 to 17 years to test whether levels of mental health and psychosocial well‐being at ages 11 and 14 years predict the likelihood of overweight and obesity reversal by age 17 years.

## METHODS

The analysis approach for this study was preregistered at the following link: https://doi.org/10.17605/OSF.IO/QMHXW

### Study design and data

We used data from the MCS, a nationally representative longitudinal birth cohort of young people born in 2000 through 2002 [26]. We selected Sweeps 5 (age 11 years; 2012–2013) and 6 (age 14 years; 2015–2016) as different study baselines to assess a range of psychological well‐being‐related measures in predicting weight changes from baseline to follow‐up at Sweep 7 (age 17 years; 2018–2019). We excluded participants with normal weight at baseline and underweight at baseline and follow‐up. The maximum analytical sample sizes varied by the following study baselines: *n* = 4556 (age 11 years, 6‐year follow‐up); and *n* = 3791 (age 14 years, 3‐year follow‐up). Ethics approval for the MCS was obtained from the National Health Service Research Ethics Committee and the National Research Ethics Service. Informed consent was obtained from caregivers when children were ages up to 14 years, and, at age 17 years, the participants or cohort members provided verbal informed consent.

### Variables

#### Weight status

We used the British 1990 child growth reference population (UK90) to standardize BMI in kilograms per meters squared into *z* scores and then define BMI categories (see online Supporting Information for full information). Weight changes from baseline to follow‐up were presented as reversal versus persistence of overweight or obesity and residualized‐change BMI *z* scores. In our study, the reversal of overweight and obesity refers to a change across survey waves from having overweight or obesity at baseline to having normal weight at follow‐up. Therefore, the term “overweight and obesity reversal” does not necessarily indicate a return to a prior state of normal weight, as some participants may have never been classified as having normal weight previously.

#### Psychological well‐being‐related measures

Table 1 presents psychological well‐being‐related measures available at baselines (ages 11 and 14 years; see online Supporting Information for full information on how psychological well‐being‐related measures were assessed). We transformed all continuous psychological well‐being‐related measures into *z* scores to allow for comparison. Because the psychological well‐being‐related measures that were examined had overlap and were likely to be conceptually related, we used exploratory factor analysis (EFA). EFA identified two distinct factors across baselines. These were best characterized as the following: 1) caregiver‐reported child mental health (internalizing and externalizing symptoms); and 2) child‐reported psychosocial well‐being (self‐esteem, depressive symptoms, life satisfaction, appearance satisfaction, and peer bullying; Table 1; Supporting Information). Individual psychological well‐being‐related measures loaded onto their corresponding indexes with factor loadings ranging from 0.47 to 0.85 without cross loadings as no measures loaded at ≥0.32 on both factors or indexes. In addition, indexes of caregiver‐reported child mental health and child‐reported psychosocial well‐being had acceptable internal consistency with Cronbach α of 0.65 and 0.81, respectively (online Supporting Information).

**TABLE 1:** Psychological well‐being related measures in MCS.

Psychological well‐being‐related measures	Baselines		Indexes of psychological well‐being‐related measures
	Sweep 5, Age 11 y	Sweep 6, Age 14 y	
Internalizing symptoms (SDQ)	Caregiver‐report	Caregiver‐report	Caregiver‐reported child mental health
Externalizing symptoms (SDQ)	Caregiver‐report	Caregiver‐report	Caregiver‐reported child mental health
Self‐esteem (Rosenberg Self‐Esteem Scale)	Self‐report	Self‐report	Child‐reported psychosocial well‐being
Depressive symptoms (using 3 items from the questionnaire in Sweep 5 and using the Short Mood and Feelings Questionnaire in Sweep 6)	Self‐report	Self‐report	Child‐reported psychosocial well‐being
Life satisfaction or happiness with life	Self‐report	Self‐report	Child‐reported psychosocial well‐being
Appearance satisfaction	Self‐report	Self‐report	Child‐reported psychosocial well‐being
Peer bullying victimization	Self‐report	Self‐report	Child‐reported psychosocial well‐being
Callous/unemotional traits (Inventory of Callous‐Unemotional Traits)	Self‐report		NA
Online bullying victimization		Self‐report	NA
Social support (Young Person Social Provisions Scale)		Self‐report	NA

1 2

To create the indexes, we recoded negative psychological well‐being‐related measures to indicate a more positive outcome by a higher score and transformed them into *z* scores. For each distinct factor, average values of corresponding standardized psychological well‐being‐related measures were calculated and then restandardized to generate an index with a mean of zero and a standard deviation (SD) of one. Better caregiver‐reported child mental health and child‐reported psychosocial well‐being were both indicated by higher scores.

#### Covariates

Covariates included sex (male or female), ethnicity (White or non‐White), and family structure (one‐ or two‐caregiver family homes). Socioeconomic status (SES) indicators such as caregiver educational and occupation groups and family income were also controlled (online Supporting Information). Because early puberty onset is associated with both mental health problems and changes in body weight [10, 27], we controlled for baseline pubertal status (age 11 or 14 years). For age 11 years as the baseline, we also controlled for changes in pubertal status from ages 11 to 14 years (online Supporting Information).

### Data analysis

#### Main analyses

We examined two indexes of psychological well‐being‐related measures, i.e., caregiver‐reported child mental health and child‐reported psychosocial well‐being, in predicting changes in overweight and obesity status from baselines (ages 11 and 14 years) to follow‐up (age 17 years) for our primary analyses. Weight changes were presented as reversal and persistence. Because indexes of psychological well‐being‐related measures were distinct factors from EFA, we fitted them together in the same regression model, to examine independent associations with outcomes, controlling for covariates. Findings were presented as odds ratio (OR) along with 95% confidence interval (CI) and *p* values.

To estimate the magnitude of change in BMI levels associated with psychological well‐being‐related measures, we also examined residualized‐change BMI *z* scores, i.e., continuous change as opposed to categorical. We estimated residual values from regressing follow‐up BMI *z* scores by baseline BMI *z* scores [28]. Linear regression was used to assess the association between indexes of psychological well‐being‐related measures and residualized‐change BMI *z* scores. Findings were presented as regression coefficient (β) along with 95% CI and *p* value.

Multiple imputation by chained equations (MICE) was used to address missing observations. Following imputation, data sets were set up for complex survey design analysis (online Supporting Information). For the main analyses mentioned earlier, significance level was set at *p* < 0.05. For the following sensitivity/additional analyses, we controlled the false discovery rate using a Benjamini‐Hochberg adjustment method [29].

#### Sensitivity/additional analyses

For regression models predicting the categorical outcome (reversal vs. persistence), we conducted sensitivity analysis by adjusting BMI *z* scores at baseline. We also fitted separate regression analyses to examine the role of individual psychological well‐being‐related measures, as opposed to the two indexes in the primary analyses, in predicting changes in weight status across the different study baselines. Furthermore, we assessed the interactions between the two indexes of psychological well‐being‐related measures and an index of SES to examine whether associations differed based on SES. Interactions between indexes of psychological well‐being‐related measures and sex were also examined. We also conducted some non‐preregistered analyses. We examined whether the association between psychological well‐being‐related measures at age 11 years consistently predicted the outcomes when measured at age 14 years (as opposed to age 17 years in the main analyses). In addition, we conducted an interaction analysis between psychological well‐being‐related measures and timing of measures, i.e., age 11 versus 14 years, in predicting overweight and obesity reversal at the same follow‐up at age 17 years in which analytical sample sizes from both baselines were combined (online Supporting Information).

## RESULTS

There were similar numbers of female and male individuals across study baselines (Table 2). Most participants were White and lived with two caregivers. Among participants with overweight or obesity at baselines (age 11 or 14 years), around 16% (participants with overweight and obesity accounted for 11% and 5% at age 11 years and 12% and 4% at age 14 years, respectively) were classified as having normal weight at follow‐up (age 17 years).

**TABLE 2:** Baseline characteristics of participants with overweight and obesity.

	Age 11 y		Age 14 y	
	*n* = 4556	Weighted %	*n* = 3791	Weighted %
Sex				
Male	2400	53.64	1870	51.28
Female	2156	46.36	1921	48.72
Ethnicity				
White	3683	81.97	2949	78.38
Non‐White	873	18.03	834	21.38
Missing	0	0	8	0.24
Family structure				
Two‐caregiver family	3349	71.00	2762	68.03
One‐caregiver family	1207	29.00	1027	31.97
Caregiver education				
No qualification	369	8.77	245	8.66
Overseas qualification	110	2.43	97	2.73
NVQ level 1	232	5.70	161	5.42
NVQ level 2	1034	24.85	785	24.34
NVQ level 3	739	16.15	586	15.41
NVQ level 4	1523	31.11	1325	30.76
NVQ level 5	526	10.37	577	11.94
Missing	23	0.62	15	0.72
Caregiver occupation				
Not in work	998	23.77	740	22.61
Semi‐routine and routine	707	15.95	586	18.21
Low supervisory, technical	209	4.54	193	5.23
Small employers, self‐employed	453	10.42	354	9.56
Intermediate	530	11.18	470	11.73
Managerial, professional	1647	33.78	1448	32.67
Missing	12	0.35	0	0
Family income				
Bottom	926	20.78	736	21.98
Second	978	22.33	706	21.15
Third	1030	21.40	809	21.36
Fourth	920	19.20	820	18.77
Top	702	16.28	717	16.64
Missing	0	0	3	0.08
Changes in weight status to age 17 y				
Persistence	2138	44.88	2235	56.25
Reversal	758	16.29	603	15.85
Missing	1660	38.84	953	27.90

*Note*: For age 11 years as baseline and age 17 years as follow‐up, the weighted proportions of participants with reversal and persistence were 26.63% (17.77% with overweight and 8.86% with obesity at baseline) and 73.37%, respectively, when participants with missing information on the outcome were not included in the calculation.

For age 14 years as baseline and age 17 years as follow‐up, the weighted proportions of participants with reversal and persistence were 21.99% (16.96% with overweight and 5.03% with obesity at baseline) and 78.01%, respectively, when participants with missing information on the outcome were not included in the calculation.

Abbreviation: NVQ, National Vocational Qualification.

A one‐SD increase in indexes of caregiver‐reported child mental health and child‐reported psychosocial well‐being at age 11 years was associated with increased odds of reversal (vs. persistence) by 16% (OR = 1.16, 95% CI: 1.03 to 1.29) and 29% (OR = 1.29, 95% CI: 1.15 to 1.44), respectively, at age 17 years (Table 3). When baseline BMI *z* scores were also controlled, only the association between child‐reported psychosocial well‐being and reversal of overweight and obesity remained statistically significant (Table  S1). Both greater caregiver‐reported child mental health (β = −0.08, 95% CI: −0.13 to −0.03) and child‐reported psychosocial well‐being (β = −0.07, 95% CI: −0.11 to −0.03) were associated with decreasing BMI *z* scores from ages 11 to 17 years. However, neither index was found to predict reversal versus persistence or BMI *z* score changes between ages 14 and 17 years. Additional analyses showed no evidence that the association between psychological well‐being and changes in overweight and obesity status or BMI *z* score levels was moderated by SES and sex (Tables S2 and S3, respectively).

**TABLE 3:** Associations between indexes of psychological well‐being‐related measures and weight changes at follow‐up of age 17 years.

Psychological well‐being‐related measures	Baseline: Age 11 y (*n* = 4556)						Baseline: Age 14 y (*n* = 3791)					
	Reversal vs. persistence of overweight and obesity by age 17 y^a^			Residualized‐change scores from ages 11 to 17 y^a^			Reversal vs. persistence of overweight and obesity by age 17 y^b^			Residualized‐change scores from ages 14 to 17 y^b^		
	OR	95% CI	*p* value	β	95% CI	*p* value	OR	95% CI	*p* value	β	95% CI	*p* value
Caregiver‐reported child mental health	1.16	1.03 to 1.29	0.013	−0.08	−0.13 to −0.03	0.001	1.05	0.92 to 1.20	0.441	−0.02	−0.07 to 0.02	0.298
Child‐reported psychosocial well‐being	1.29	1.15 to 1.44	<0.001	−0.07	−0.11 to −0.03	0.002	1.07	0.94 to 1.23	0.304	−0.01	−0.04 to 0.03	0.791

*Note*: Indexes of psychological well‐being‐related measures are expressed as *z* scores (mean = 0; SD = 1).

Abbreviation: OR, odds ratio.

^a^ Both indexes were fitted in the same regression model, adjusting for sex, ethnicity, family structure, caregiver education, caregiver employment, family income, pubertal status at age 11 years, and changes in pubertal status from ages 11 to 14 years.

^b^ Both indexes were fitted in the same regression model, adjusting for sex, ethnicity, family structure, caregiver education, caregiver employment, family income, and pubertal status at age 14 years.

We reasoned that the more pronounced associations between psychological well‐being‐related measures and the outcomes when age 11 years was the baseline may be due to longer versus shorter follow‐up periods (6 vs. 3 years). Therefore, we conducted an exploratory analysis (not preregistered) to examine whether psychological index measures at age 11 years consistently predicted outcomes with the same follow‐up period (3 years), using age 14 years, as opposed to age 17 years, as follow‐up. A higher index of child‐reported psychosocial well‐being at age 11 years was associated with overweight and obesity reversal (vs. persistence) and residualized‐change BMI *z* scores from ages 11 to 14 years (Table S4). The association between an index of caregiver‐reported child mental health and residualized‐change BMI *z* scores was no longer statistically significant when adjusting for multiple comparisons.

An index of child‐reported psychosocial well‐being at age 11 years, but not age 14 years, was consistently associated with weight changes across different follow‐up periods, and this may indicate that psychological well‐being‐related measures at age 11 years are more important than those at age 14 years in predicting weight changes. Therefore, we formally examined statistical evidence for differences in predictive ability based on when psychological well‐being‐related measures were collected, computing interactions between the indexes and baselines (age 11 vs. 14 years) in predicting weight changes for the same follow‐up (age 17 years) by combining analytical sample sizes from both baselines (*n* = 8347). We found statistically significant interactions, indicating a noticeable larger effect size for age 11 versus 14 years, between the index of child‐reported psychosocial well‐being and baseline in the prediction of reversal versus persistence and changes in BMI *z* scores (Table S5), which provides further evidence of psychological well‐being‐related measures at age 11 versus 14 years being more strongly associated with weight‐change outcomes.

Results from additional analyses largely supported the primary analyses because most individual psychological well‐being‐related measures that defined indexes of caregiver‐reported child mental health (internalizing and externalizing symptoms) and child‐reported psychosocial well‐being (self‐esteem, depressive symptoms, life satisfaction, appearance satisfaction, and peer bullying) when examined separately from age 11 years, not age 14 years, statistically significantly predicted the outcomes at age 17 years (Table 4). The associations between individual measures and weight changes were also pronounced when age 11 years was used as the baseline, irrespective of the follow‐up periods (i.e., 3 and 6 years) examined (e.g., internalizing symptoms, life satisfaction; Table S6). Similar to the main analyses examining the indexes, a number of individual measures (e.g., depression, life satisfaction) had stronger associations with weight outcomes measured at age 11 years as opposed to age 14 years (Table S7).

**TABLE 4:** Associations between individual psychological well‐being related measures and weight changes at follow‐up of age 17 years.

Psychological well‐being‐related measures	Baseline: Age 11 y (*n* = 4556)						Baseline: Age 14 y (*n* = 3791)					
	Reversal vs. persistence of overweight and obesity by age 17 y^a^			Residualized‐change scores from ages 11 to 17 y^a^			Reversal vs. persistence of overweight and obesity by age 17 y^b^			Residualized‐change scores from ages 14 to 17 y^b^		
	OR	95% CI	*p* value	β	95% CI	*p* value	OR	95% CI	*p* value	β	95% CI	*p* value
Internalizing symptoms	0.83	0.75 to 0.93	0.002*	0.07	0.03 to 0.11	<0.001*	0.92	0.81 to 1.04	0.166	0.02	−0.02 to 0.06	0.374
Externalizing symptoms	0.84	0.75 to 0.94	0.002*	0.09	0.05 to 0.13	<0.001*	0.95	0.83 to 1.08	0.405	0.03	−0.01 to 0.07	0.091
Self‐esteem	1.16	1.03 to 1.30	0.012*	−0.03	−0.07 to 0.01	0.100	1.05	0.92 to 1.19	0.502	0.01	−0.03 to 0.04	0.673
Depressive symptoms	0.78	0.70 to 0.86	<0.001*	0.09	0.06 to 0.13	<0.001*	0.99	0.88 to 1.11	0.808	0.02	−0.02 to 0.06	0.259
Life satisfaction	1.22	1.08 to 1.39	0.003*	−0.06	−0.10 to −0.02	0.002*	0.96	0.85 to 1.09	0.508	0.01	−0.03 to 0.04	0.714
Appearance satisfaction	1.21	1.08 to 1.35	0.001*	−0.04	−0.09 to 0.00	0.064	1.12	1.00 to 1.27	0.059	0.00	−0.04 to 0.04	0.928
Peer bullying	0.83	0.75 to 0.92	<0.001*	0.10	0.06 to 0.14	<0.001*	0.87	0.76 to 0.99	0.031	0.03	−0.00 to 0.07	0.076
Callous/unemotional traits	0.84	0.75 to 0.94	0.003*	0.04	−0.01 to 0.08	0.083	NA			NA		
Online bullying	NA						1.01	0.90 to 1.13	0.918	0.02	−0.02 to 0.06	0.287
Social support	NA						0.94	0.83 to 1.07	0.328	0.02	−0.02 to 0.06	0.356

*Note*: All individual psychological well‐being‐related measures are expressed as *z* scores (mean = 0; SD = 1).

Abbreviations: NA, not applicable (i.e., psychological measure was not available); OR, odds ratio.

^a^ Separate regression models were developed for each individual psychological well‐being‐related measure, adjusting for sex, ethnicity, family structure, caregiver education, caregiver employment, family income, pubertal status at age 11 years, and changes in pubertal status from ages 11 to 14 years.

^b^ Separate regression models were developed for each individual psychological well‐being‐related measure, adjusting for sex, ethnicity, family structure, caregiver education, caregiver employment, family income, and pubertal status at age 14 years.

* *p* value remained statistically significant after correcting multiple comparisons using Benjamini‐Hochberg adjustment method (see Tables S8 and S9).

## DISCUSSION

Better child mental health and psychosocial well‐being at age 11 years were prospectively and independently associated with increased odds of reversal (vs. persistence) of overweight and obesity and a reduction in BMI *z* scores at age 17 years. Across analyses, psychological measures only predicted weight‐change outcomes when measured at age 11 years, and not at age 14 years. This finding highlights the possibility of an age‐specific, sensitive period when psychological measures have a particularly pronounced relationship with reversal of childhood overweight and obesity.

Using a nationally representative birth cohort study of UK children, the MCS, our study extends past research using the same data on this topic. Two previous studies have analyzed MCS data to examine the association between BMI and psychological well‐being from childhood to adolescence and found that the association between both is stronger as children get older [25] and that a high BMI growth trajectory is associated with worse psychosocial well‐being [30]. Another study suggested that self‐esteem and appearance satisfaction may explain the link between childhood BMI and adolescent mental health [31]. No studies, to our knowledge, have examined whether a range of psychological well‐being‐related measures explains why children reversed overweight and obesity status by the end of adolescence whereas others do not. Therefore, findings from our study represent a new contribution to the literature.

Understanding why and how psychological well‐being relates to child overweight and obesity reversal will now be important. Poorer psychological well‐being may be associated with persistence of heavier body weight through chronic elevation of cortisol. Hypothalamic‐pituitary‐adrenocortical axis hyperactivation has been suggested to play a role in increasing appetite and fat accumulation [17, 18, 32]. In support of this, cortisol reactivity has also been found to mediate the association between depression and increased BMI values in girls [33]. Alternatively, behavioral responses to cope with poorer well‐being and stress may also contribute to the persistence of overweight and obesity. Weight‐related stigma has been shown to be associated with unhealthy eating and reduced physical activity [12, 13, 34]. Likewise, other psychological well‐being‐related measures such as mental health difficulties (e.g., internalizing symptoms) and depressive symptoms may contribute to the persistence of overweight and obesity through unhealthy diet and physical inactivity [14, 15, 16]. In contrast, positive psychological well‐being in children with overweight or obesity may be associated with a reduction in ghrelin and cortisol levels [35] and greater adoption of health‐promoting behaviors [36] that can facilitate changes to normal weight.

In this present study, the longitudinal associations between psychological well‐being‐related measures and weight changes were more salient for psychological well‐being‐related measures when collected at age 11 years rather than age 14 years. This indicates that late childhood or early adolescence (i.e., age 11 years) may be a sensitive period for psychological well‐being in predicting subsequent body weight trajectories; however, it is unclear why this is the case, and further research is required to confirm and understand this finding. Early adolescence may be a critical period for some children because they will experience mental health difficulties for the first time [37, 38]. In the UK context, children at age 11 years experience psychosocial changes due to the transition from primary to secondary school that may have impacts on their present and future mental health [39]. Concerns over peer bullying and impact on existing friendships are major sources of worries in children regarding their transition from primary to secondary school [40]. Psychological outcomes associated with this transition may further be associated with weight changes through physiological and unhealthy behavioral coping mechanisms. For example, current evidence indicates that the transition from primary to secondary school is associated with more sedentary behavior and less consumption of fruit and vegetables [41], which may play an important role in maintaining current overweight and obesity status for some children.

Early puberty onset has been associated with experiencing mental health problems and changes in body weight [10, 27]. To address this, our analyses controlled for baseline pubertal status (age 11 or 14 years) and changes in pubertal status from ages 11 to 14 years (defined as “yes” if puberty was reported at age 14 years, not at age 11 years; online Supporting Information). However, pubertal status measurement and estimation of timing of puberty onset may have been imprecise owing to being self‐reported; therefore, further research may benefit from addressing these limitations.

Because the extensive psychological well‐being‐related measures that were examined were shown to be interrelated and loaded onto two overarching and statistically distinct factors, we examined the extent to which overall scores on these overarching factors predicted weight‐change outcomes. Higher scores on the index measurement of caregiver‐reported child mental health may be best described as characterizing child presentation of mental health difficulties (i.e., internalizing and externalizing symptoms), whereas higher scores on the index of child‐reported psychosocial well‐being relate to feeling more negatively about oneself and not fitting in (e.g., self‐esteem, victimization). The potential strength of the index approach that we adopted is underlined by the observation that the index measure of child‐reported psychosocial well‐being tended to be a stronger predictor of weight‐change outcomes than the index measure of caregiver‐reported child mental health and the individual measures of psychosocial well‐being‐related measures that were examined in isolation (e.g., better child‐reported psychosocial well‐being was associated with a 29% increased odds of reversal of childhood overweight or obesity). A number of studies have examined the associations between separate psychological well‐being‐related measures and weight changes in childhood [25, 30, 31]. However, the results of the present study suggest that some measures are likely to be strongly interrelated and may be better considered in concert when characterizing overall psychosocial well‐being.

We found no evidence that the associations between psychological well‐being‐related measures and weight changes were modified by SES. Given that both poorer mental health and adverse psychosocial outcomes are more common among children from lower SES backgrounds and that childhood overweight and obesity development is more prevalent in families with lower SES [42], this suggests that mental health and psychosocial well‐being could be important factors explaining why tracking of child overweight and obesity into adulthood is more pronounced in families with lower SES. We examined household SES, but not local area SES (e.g., the level of deprivation of the neighborhoods where cohort members lived). Local area SES may be important to consider in future research. For example, living in a more deprived neighborhood may result in exposure to more obesogenic environmental factors [43], which subsequently shape the association between psychological well‐being and weight changes. Further research formally examining this would now be informative.

This is the first study, to our knowledge, to comprehensively examine an extensive range of psychological well‐being‐related measures in predicting childhood overweight and obesity reversal. Additional strengths include our wave‐by‐wave analyses to examine potential sensitive periods for psychological well‐being in predicting future weight changes. However, the study has some limitations. We defined ethnicity as White and non‐White because the majority of our sample was White, and only a small proportion of participants were from ethnic minority groups. Therefore, testing the association in more ethnically diverse samples will now be important. The psychological well‐being‐related measures included appearance satisfaction and peer bullying but did not refer specifically to body weight. Although we did not include experience of teasing and bullying related specifically to weight in the current study, weight‐based teasing and bullying are commonly experienced by children and adolescents with overweight and obesity [44], and these experiences are associated with lower appearance satisfaction [45], as well as several other adverse health outcomes [34]. Future research will benefit from directly assessing the experience of weight‐based teasing and bullying. Although BMI *z* scores are widely used, this measure may not be sensitive to characterize children with very high levels of adiposity and does not account for body composition. Exploring whether the same psychological well‐being‐related measures predict other markers that may better characterize child adiposity (e.g., body fat percentage) is now warranted. Analyses accounted for pubertal status at baseline and onset of puberty by follow‐up (for age 14 years), but measurement was reliant on indirect parent reporting, which may be prone to some bias. Nonetheless, results were similar in male and female individuals, and results from models including versus excluding pubertal status controls produced comparable results. Furthermore, our analysis was limited to reversal (vs. persistence) of childhood overweight and obesity; therefore, it would now be valuable to examine whether the same psychological well‐being‐related measures are associated with the initial development of overweight and obesity in childhood or its initial development in adolescence, i.e., with no previous history of having childhood overweight and obesity. In addition, future studies exploring the role of psychological well‐being in predicting the adult reversal of overweight and obesity may now also be valuable.

## CONCLUSION

Better psychosocial well‐being at age 11 years is associated with increased likelihood of reversing childhood overweight and obesity by age 17 years. Late childhood/early adolescence may be a sensitive period in which psychological well‐being has a particularly pronounced prospective relationship with body weight trajectories.

## AUTHOR CONTRIBUTIONS

All of the authors were involved in conceptualizing the study. I Gusti Ngurah Edi Putra carried out the initial analysis and drafted the initial manuscript. Michael Daly and Eric Robinson critically reviewed and revised the manuscript. All authors approved the submitted and published versions of the manuscript.

## FUNDING INFORMATION

This work received funding from the Economic and Social Research Council (ESRC), which is part of UK Research and Innovation (UKRI; ES/V017594/1). The funder had no role in the study design, data analysis, interpretation of the findings, manuscript preparation, approval of the manuscript, or decision to publish.

## CONFLICT OF INTEREST STATEMENT

The authors declared no conflict of interest.

## Supporting information


**Data S1:** Supplementary Information.

## Data Availability

Data are available online (https://doi.org/10.5255/UKDA-Series-2000031) with the permission of the data custodian.

## Footnotes

1
*Note*: Exploratory factor analysis (EFA) was used to identify the underlying relationships among psychological well‐being‐related measures. We used varimax rotation to simplify data structure and identify separate factors. Two psychological well‐being‐related measures (internalizing and externalizing symptoms) reported by caregivers constituted one factor, and the remaining five measures (self‐esteem, depression, life satisfaction, appearance satisfaction, and peer bullying) self‐reported by cohort members defined another factor. See online Supporting Information for full information on scales or questions used to quantify psychological well‐being‐related measures and the development of indexes.

2 Abbreviations: NA, not applicable (not included in the development of any index because it was unavailable at both baseline waves and not included in factor analysis); SDQ, Strengths and Difficulties Questionnaire.
