# Supplementary material for: Psychological well‐being and the reversal of childhood overweight and obesity in the UK: a longitudinal national cohort study
Source: Obesity (Silver Spring). 2024 Oct 7;32(12):2354–63. doi: 10.1002/oby.24147 (PMC11589541; doi:10.1002/oby.24147)
Supplement: Supplementary file 1 — Data S1: Supplementary Information. [file OBY-32-2354-s001.pdf]

Supplementary materials

Supplementary information

Weight status

Body mass index (BMI) in kg/m^2 was assessed by trained interviewers using standardised instruments (see [1]). Participants with outlier values of BMI (< 10 or > 50) were omitted (e.g., as in [2]). Following a previous study [3], we used the British 1990 child growth reference population (UK90 reference) [4] which is more appropriate for the UK context to standardise BMI into z-scores and define the BMI category. We used 'zanthro' command in STATA to standardise BMI following the UK90 reference in which information on cohort members' sex and age at the time of assessment (or follow-up interviews) were taken into account [5]. BMI category (underweight, normal weight, overweight, obesity) based on the UK90 reference was available in the datasets of Sweeps 6 (age 14) and 7 (age 17), but not in Sweep 5 (age 11). Therefore, for Sweep 5 (age 11), we transformed BMI z-scores into percentiles using 'normal' command in STATA (see [6]), and then the BMI category was determined based on UK90 percentile or centile cut-offs [7, 8].

Psychological well-being related measures

Internalising and externalising symptoms

In both Sweeps 5 and 6, the Strengths and Difficulties Questionnaire (SDQ) [9] was used to assess internalising and externalising symptoms. This tool has been widely used to screen for probable mental health problems in children across different study settings [10, 11, 12]. SDQ comprises 25 items (e.g., "Many worries, often seems worried", "Often fights with other children or bullies them") rated on a 3-point Likert scale. These items are evenly distributed across five subscales (five items for each subscale), namely emotional symptoms, peer problems, conduct problems, hyperactivity, and prosocial behaviour. Caregivers' responses as "not true", "somewhat true", and "certainly true" to the items of four deficit-focused subscales were scored as 0, 1, and 2, respectively, and scores were reverse coded for the prosocial behaviour subscale. Therefore, each subscale had a total score ranging from 0 to 10 with a higher score indicating more negative problems, but more positive for prosocial behaviour. The total scores of emotional symptoms and peer problems were added together to compute scores of internalising symptom, whilst the other two problem subscales, conduct problems and hyperactivity, were used to define externalising symptom (e.g., as in [13]). The total score of each symptom ranged from 0 to 20.

Self-esteem

A shortened and adapted version of the Rosenberg Self-Esteem Scale [14] that consists of five items was administered to the cohort members to assess their self-esteem (e.g., "On the whole, I am satisfied with myself", "I feel that I have a number of good qualities") in both Sweeps 5 and 6. Participants' responses on a 4-point Likert scale (from 1= "strongly disagree" to 4= "strongly agree") were summed to provide a total score ranging from 1 to 20 (e.g., as in [15, 16]). A higher total score indicated greater self-esteem.

Callous/unemotional traits

A shortened version of the youth version of the Inventory of Callous-Unemotional Traits (ICU youth version) [17] was self-completed by the participants in Sweep 5. This adapted tool consists of four items

representing three subscales of the ICU youth version: callousness (one item), uncaring (two items) and unemotional (one item) (e.g., "I feel bad or guilty when I have done something wrong", "I do not show my emotions to others"). A summary score was generated by adding together responses on a 4-point Likert scale (from "not at all true" = 0 to "definitely true" = 3). A greater level of callous or unemotional traits was indicated by a higher score on a scale of 0 to 12 for a total or summary score.

Depressive symptoms

Following a previous study using the Millenium Cohort Study (MCS) data [18], the following three items that were self-completed by the participants were used to define depressive symptoms in Sweep 5: "In the last four weeks, how often did you feel sad?", "get worried about what would happen to you?", and "feel afraid or scared?". Responses to all the items on a 5-point Likert scale (from "never" = 1 to "almost always" = 5) were summed to create a total score ranging from 1 to 15 with a higher score indicating greater depressive symptoms. This three-item scale was reported to have good internal consistency [18].

In Sweep 6, depressive symptoms were assessed using 13 items from the Short Mood and Feelings Questionnaire (SMFQ) [19]. Participants self-rated on a 3-point Likert scale ranging from "not true" = 1 to "true" = 3 regarding how they felt in the past two weeks (e.g., "I felt miserable or unhappy", "I didn't enjoy anything at all"). A total score ranging from 1 to 39 was computed by adding together responses from all the items with a higher score indicating higher depressive symptoms (e.g., as in [18]).

Life satisfaction

Following previous studies [15, 20], five items reflecting how the participants perceived their life, family, friends, and school were used to quantify overall life satisfaction or happiness with life in both Sweeps 5 and 6. Their responses to a 7-point Likert scale ranging from "not at all happy" = 1 to "completely happy" = 7 were totalled. A higher score on a scale of 1 to 35 for a total score indicated greater life satisfaction.

Appearance satisfaction

Previous studies that examined psychological correlates of body weight status used a single item on how participants felt about the way they 'look' from the happiness scale as a proxy of 'body image' or 'body satisfaction' [2, 16, 18]. In both Sweeps 5 and 6, we used this measure to indicate appearance satisfaction instead of body satisfaction as the item did not explicitly indicate 'body'. A score ranged from 1 to 7 with a higher score indicating greater appearance satisfaction.

Bullying victimisation

In Sweep 5, participants were asked an item regarding their experience of being bullied by peers: "How often do other children hurt you or pick on you on purpose?". Responses were recorded on a 6-point Likert scale from "most days" = 1 to "never" = 6. In Sweep 6, another item on online bullying was administered to the participants: "How often have other children sent you unwanted or nasty emails, texts or messages or posted something nasty about you on a website?" with the same options of answers. Following a previous approach [21], scores were reverse coded to indicate more frequent experience of bullying by a higher value and these two items of bullying experience in Sweep 6 were treated as separate variables. In comparison to our pre-registered protocol (https://doi.org/10.17605/OSF.IO/QMHXW), we decided to not include bullying experience from sibling reported in both Sweeps 5 and 6 as not all the participants reported having a sibling (7%), resulting in more missing observations and not appropriate to be addressed by multiple imputations (see 'Data analysis').

Social support

In Sweep 6, perceived social support in young people was measured using three items from the Young Person Social Provisions Scale [22]. Participants were asked to think about their current relationships with other people (e.g., "I have family and friends who help me feel safe, secure and happy", "There is someone I trust whom I would turn to for advice if I were having problems"). Their responses on a 3-point Likert scale (from "not true at all" = 1 to "very true" = 3) across all items were totalled, resulting in a sum score ranging from 1 to 9 with a higher score indicating greater social support (e.g., as in [21]).

The development of indexes of psychological well-being related measures

Exploratory factor analysis (EFA) [23] was used to identify the underlying relationships among psychological well-being related measures. Compared to the pre-registered protocol, we only included psychological well-being related measures that were available at both baselines (n = 7) for EFA so that this would allow for comparison of these psychological measures between different baselines (ages 11 vs. 14) in predicting weight changes. Findings from EFA showed that two factors had Eigenvalue > 1.0. We then used varimax rotation to simplify data structure and identify separate factors [24]. Two psychological well-being related measures: internalising and externalising symptoms reported by caregivers constituted one factor, and the remaining five measures (self-esteem, depression, life satisfaction, appearance satisfaction, and peer bullying) self-reported by cohort members defined the other factor.

Based on these two distinct factors, indexes were then developed and termed caregiver-reported child mental health and child-reported psychosocial well-being. We first re-coded negative psychological well-being related measures (internalising and externalising symptoms, depressive symptoms, peer bullying reversed) to indicate a more positive outcome by a higher score. Next, all the psychological well-being related measures were transformed into z-scores to allow comparison across different metrics. Average values of corresponding standardised individual psychological well-being related measures were calculated and then re-standardised to generate an index with a mean of zero and a standard deviation of one. To test convergent validity, Spearman's rank correlation was used to examine correlations between indexes and individual psychological well-being related measures [25]. There were strong correlations between individual psychological well-being related measures and their related index. Using Cronbach's Alpha to determine the internal consistency reliability [23], indexes of caregiver-reported child mental health and child-reported psychosocial well-being were observed with acceptable internal consistency (Cronbach's Alpha of 0.65 and 0.81, respectively) [26].

Item loadings for psychological well-being related measures

Psychological well-being related measures	Item loadings	
	Factor 1	Factor 2
Internalising symptoms	0.162	**0.823**
Externalising symptoms	-0.001	**0.854**
Self-esteem	**0.831**	0.034
Depressive symptoms	**0.808**	0.157
Life satisfaction	**0.759**	0.213
Appearance satisfaction	**0.824**	-0.025
Peer bullying	**0.470**	0.286

Correlation between individual psychological well-being related measures and indexes

Psychological well-being related measures	Correlation coefficients	
	Index of caregiver-reported child mental health	Index of child-reported psychosocial well-being
Internalising symptoms	**0.798*****	0.249***
Externalising symptoms	**0.868*****	0.137***
Self-esteem	0.144***	**0.776*****
Depressive symptoms	0.181***	**0.776*****
Life satisfaction	0.263***	**0.806*****
Appearance satisfaction	0.106***	**0.788*****
Peer bullying	0.157***	**0.559*****

***p < 0.001

Covariates

Caregiver education was defined based on the highest academic or vocational qualification. We used a derived variable available in the dataset that classified caregiver education into National Vocational Qualification (NVQ) scale [27], consisting of NVQ level 1 ("Certificate of Secondary Education - CSE below grade 1; General Certificate of Secondary Education (GCSE) or O Level below grade C; The Scottish Certificate of Education (SCE) Standard, Ordinary grades below grade 3 or Junior Certificate below grade C"), level 2 ("GCSE or O Level grade A-C; SCE Standard, Ordinary grades 1-3 or Junior Certificate grade A-C"), level 3 ("A/AS/S Levels/SCE Higher; Scottish Certificate Sixth Year Studies; Leaving Certificate"), level 4 (ranging from teaching qualification below degree level to first-degree qualification), and level 5 (higher degree and postgraduate qualification, including postgraduate diplomas and certificates), with additional groups for overseas qualification and no qualification (e.g., as in [28]). National Statistics Socio-economic Classification was used to categorise occupation into five main groups (OGs) [27]: 1) managerial, administrative, and professional occupations, 2) intermediate occupations, 3) small employers and self-employed, 4) lower supervisory and technical occupations, 5) semi-routine and routine occupations, with another group for 6) unemployed (e.g., as in [29]). For both educational and occupation groups, the highest group was selected for cohort members from families with more than one caregiver (main caregiver and partner) who were from different groups (e.g., as in [28]). Equivalised household income was calculated using the Organisation for Economic Co-operation and Development (OECD) guidelines in which disposable weekly household income was adjusted for the family composition and structure [27]. We used an available variable of equivalised household income in the dataset that has been transformed into quintiles (bottom [£65.85 – £224.97], second [£224.98 – £326.92], third [£326.94 – £441.28], fourth [£441.35 – £571.04], top [£571.05 – £1162.8]) (e.g., as in [16]).

Compared to the pre-registered analysis approach, we also controlled pubertal status. Pubertal status at ages 11 or 14 was defined as whether males experienced voice deepening or females had menarche, reported by caregivers (age 11) or cohort members (age 14) following a previous approach [30]. Across all the analyses predicting weight changes at age 17, we controlled baseline pubertal status at age 11 or 14 depending on the baseline. For age 11 as the baseline (ages 14 or 17 as follow-ups), we also controlled for changes in pubertal status from age 11 to 14 (defined "yes" if participants did not have puberty at age 11, but they reported puberty at age 14) to better account for the potential influence of puberty onset during this period for which models were predicting weight changes. We did not control for pubertal status at age 17 as puberty was not asked to all the cohort members. Analyses with controls

for pubertal status produced similar sized effect estimates and results as analyses without pubertal status controls (data not shown).

Data analysis

Multiple imputation

We fitted multiple imputation by chained equations (MICE) to address missing observations and potential selection bias. Compared to single imputations, multiple predictions are created for each missing value in MICE to reduce uncertainty in the imputations, and it is very flexible for the range of different measures available in this study (e.g., continuous or categorical variables) [31]. We assumed missing at random (MAR) as some variables, including family structure and household income were found to be predictive of missingness in the dataset. Using 'mi impute chained' command in STATA, we generated 20 inputted datasets to fill in missing information in all variables, including the independent, dependent variables, and covariates (e.g., as in [32]). To improve predictions, we included some auxiliary variables (i.e., variables that are not included/examined in the analysis, but are correlated with a missing variable or missingness), such as number of people living in the household, housing tenure, neighbourhood disadvantage, children's general health, longstanding illness, main caregiver's psychological distress, general health, longstanding illness, and perception of financial difficulty, with stratum (i.e., disadvantage stratification within country) added as a covariate (e.g., as in [28]). In the imputation model, we used variables with minimum or without missing observations (e.g., sex, family structure, family income) as predictors (e.g., as in [33]). The imputation model also included baseline non-response adjusted sample weights available in MCS (see [34, 35]). Following the imputation, we used 'mi svyset' command to set up inputted datasets for complex survey design (see [34]). We then applied 'mi estimate: svy' command to run all regression models. Using the MICE approach, sample size was the same as the maximum analytical sample sizes across different baselines reported in Table 2 in the main paper. Table S10 presents associations between indexes of psychological well-being related measures and the outcomes (reversal vs. persistence; residualised change scores) using a complete-case analysis, and the findings were largely consistent with the results obtained using the MICE approach to address missing values (Table 3 in the main paper).

Sensitivity/additional analyses

For regression models predicting the categorical outcome (reversal vs. persistence), we conducted a sensitivity analysis by adjusting BMI z-scores at baseline. Cohort members with significant differences in BMI would be grouped together either as normal weight (e.g., participants with normal weight closer to underweight vs. overweight) or overweight/obesity (participants with just overweight vs. with severe obesity). Therefore, psychological well-being related measures may be associated with weight within these categories instead of comparison between categories. We aimed to examine whether the results for the main analyses above were consistent if baseline BMI z-scores were controlled.

The main analyses were replicated for examining individual psychological well-being related measures across different study baselines (Sweeps 5 and 6). We developed separate regression models for each psychological measure in predicting the outcomes (reversal vs. persistence; residualised change scores). This is because individual psychological well-being related measures were not distinct factors and may share some variances when being included in the same regression model.

As lower SES is associated with worse psychological outcomes [29] and unhealthy weight status [36] in a sample of UK children and adolescents, we also examined whether the associations between the index of psychological well-being related measures (caregiver-reported child mental health or child-reported

psychosocial well-being) and changes in weight status are contingent upon SES. Two-way interaction terms between indexes of psychological well-being related measures and an index of SES were added to the regression models in predicting the outcomes. We followed a previous study to develop an SES index variable [37] by re-standardising the average values of three standardised SES indicators (caregiver education, occupation, and household income). A higher index indicated higher overall SES.

We conducted some non-preregistered analyses. We examined the interactions between indexes of psychological well-being related measures and sex in predicting weight changes. Two-way interaction terms between indexes of psychological well-being related measures and sex (female vs. male) were added to the regression model. In addition, we examined whether the associations between psychological well-being related measures at age 11 and overweight and obesity reversal measured at age 17 (6-year follow-up) remained for a shorter follow-up (3 years). Regression models were fitted for psychological well-being related measures at age 11, using age 14 as the follow-up. Furthermore, we conducted an interaction analysis to examine differences in predictive ability based on when psychological well-being related measures were collected. For this analysis, we combined analytical sample sizes from both baselines. Two-way interaction terms between psychological well-being related measures (indexes and individual measures) and timing of measures (ages 11 vs. 14) were fitted in regression models predicting outcomes at the same follow-up at age 17 in the pooled analytical sample size (n = 8,347). To address potential correlations between observations within individuals, we incorporated the ‘vce(cluster participant_ID)’ option into the primary regression commands in STATA. This adjustment ensures that standard errors are appropriately corrected for clustering observations at the individual level. It is important to note that for this interaction analysis, the data was not structured for complex survey design analysis (without using the 'svy' command) because it was not compatible with the clustering option.

References

1. Centre for Longitudinal Studies. Millennium Cohort Study, Sixth Survey 2015-2016, User Guide (Second Edition) Centre for Longitudinal Studies, UCL; 2020. Available from: https://doc.ukdataservice.ac.uk/doc/8156/mrdoc/pdf/mcs6_user_guide_ed2_2020-08-10.pdf.
2. Kelly Y, Patalay P, Montgomery S, Sacker A. BMI Development and Early Adolescent Psychosocial Well-Being: UK Millennium Cohort Study. *Pediatrics* 2016;**138**.
3. Mead E, Batterham AM, Atkinson G, Ells LJ. Predicting future weight status from measurements made in early childhood: a novel longitudinal approach applied to Millennium Cohort Study data. *Nutrition & Diabetes* 2016;**6:** e200-e200.
4. Wright CM, Booth IW, Buckler JM, Cameron N, Cole TJ, Healy MJ*, et al.* Growth reference charts for use in the United Kingdom. *Arch Dis Child* 2002;**86:** 11-14.
5. Vidmar SI, Cole TJ, Pan H. Standardizing Anthropometric Measures in Children and Adolescents with Functions for Egen: Update. *The Stata Journal* 2013;**13:** 366-378.
6. Neuhauser HK, Büschges J, Schaffrath Rosario A, Schienkiewitz A, Sarganas G, Königstein K*, et al.* Carotid Intima-Media Thickness Percentiles in Adolescence and Young Adulthood and Their Association With Obesity and Hypertensive Blood Pressure in a Population Cohort. *Hypertension* 2022;**79:** 1167-1176.
7. Hudda MT, Nightingale CM, Donin AS, Owen CG, Rudnicka AR, Wells JCK*, et al.* Patterns of childhood body mass index (BMI), overweight and obesity in South Asian and black participants in the English National child measurement programme: effect of applying BMI adjustments standardising for ethnic differences in BMI-body fatness associations. *Int J Obes (Lond)* 2018;**42:** 662-670.
8. Centre for Longitudinal Studies. Child overweight and obesity: Initial findings from the Millennium Cohort Study Age 14 Survey: Centre for Longitudinal Studies, UCL; 2017. Available from: https://cls.ucl.ac.uk/wp-content/uploads/2017/12/MCS6-Briefing-02-Overweight-and-obesity.pdf.
9. Goodman R. The Strengths and Difficulties Questionnaire: a research note. *J Child Psychol Psychiatry* 1997;**38:** 581-586.
10. Goodman A, Goodman R. Strengths and difficulties questionnaire as a dimensional measure of child mental health. *Journal of the American Academy of Child & Adolescent Psychiatry* 2009;**48:** 400-403.
11. Hall CL, Guo B, Valentine AZ, Groom MJ, Daley D, Sayal K*, et al.* The validity of the Strengths and Difficulties Questionnaire (SDQ) for children with ADHD symptoms. *PLoS One* 2019;**14:** e0218518-e0218518.
12. Croft S, Stride C, Maughan B, Rowe R. Validity of the Strengths and Difficulties Questionnaire in Preschool-Aged Children. *Pediatrics* 2015;**135:** e1210-e1219.
13. Papachristou E, Flouri E. Distinct developmental trajectories of internalising and externalising symptoms in childhood: Links with mental health and risky behaviours in early adolescence. *Journal of Affective Disorders* 2020;**276:** 1052-1060.
14. Rosenberg M. *Society and the Adolescent Self-Image*. Princeton University Press, 1965.
15. Bannink R, Pearce A, Hope S. Family income and young adolescents' perceived social position: associations with self-esteem and life satisfaction in the UK Millennium Cohort Study. *Arch Dis Child* 2016;**101:** 917-921.
16. Creese H, Saxena S, Nicholls D, Pascual Sanchez A, Hargreaves D. The role of dieting, happiness with appearance, self-esteem, and bullying in the relationship between mental health and body-mass index among UK adolescents: a longitudinal analysis of the Millennium Cohort Study. *eClinicalMedicine* 2023;**60**.
17. Essau CA, Sasagawa S, Frick PJ. Callous-unemotional traits in a community sample of adolescents. *Assessment* 2006;**13:** 454-469.

18. Sharpe H, Fink E, Duffy F, Patalay P. Changes in peer and sibling victimization in early adolescence: longitudinal associations with multiple indices of mental health in a prospective birth cohort study. *Eur Child Adolesc Psychiatry* 2022;**31:** 737-746.
19. Angold A, Costello EJ, Messer SC, Pickles A. Development of a short questionnaire for use in epidemiological studies of depression in children and adolescents. *International Journal of Methods in Psychiatric Research* 1995;**5:** 237-249.
20. Booker CL, Skew AJ, Sacker A, Kelly YJ. Well-Being in Adolescence—An Association With Health-Related Behaviors: Findings From Understanding Society, the UK Household Longitudinal Study. *The Journal of Early Adolescence* 2013;**34:** 518-538.
21. Yang K, Petersen KJ, Qualter P. Undesirable social relations as risk factors for loneliness among 14-year-olds in the UK: Findings from the Millennium Cohort Study. *International Journal of Behavioral Development* 2020;**46:** 3-9.
22. Cutrona CE, Russell DW. The provisions of social relationships and adaptation to stress. *Advances in Personal Relationships* 1987;**1:** 37-67.
23. Fenn J, Tan C-S, George S. Development, validation and translation of psychological tests. *BJPsych Advances* 2020;**26:** 306-315.
24. Osborne JW. What is Rotating in Exploratory Factor Analysis? *Practical Assessment, Research, and Evaluation* 2015;**20**.
25. Sacre H, Haddad C, Hajj A, Zeenny RM, Akel M, Salameh P. Development and validation of the Socioeconomic Status Composite Scale (SES-C). *BMC Public Health* 2023;**23:** 1619.
26. Taber KS. The Use of Cronbach's Alpha When Developing and Reporting Research Instruments in Science Education. *Research in Science Education* 2018;**48:** 1273-1296.
27. Rosenberg R, Atkinson M. MCS5: Guide to Derived Variables Centre for Longitudinal Studies, UCL; 2015. Available from: https://cls.ucl.ac.uk/wp-content/uploads/2017/07/mcs5_Derived_Variables.pdf.
28. Staatz CB, Kelly Y, Lacey RE, Hardy R. Area-level and family-level socioeconomic position and body composition trajectories: longitudinal analysis of the UK Millennium Cohort Study. *The Lancet Public Health* 2021;**6:** e598-e607.
29. Hazell M, Thornton E, Haghparast-Bidgoli H, Patalay P. Socio-economic inequalities in adolescent mental health in the UK: Multiple socio-economic indicators and reporter effects. *SSM - Mental Health* 2022;**2:** 100176.
30. Pongiglione B, Fitzsimons E. Overweight and obesity in childhood and adolescence: findings from the UK Millennium Cohort Study, up to age 14. *Longitudinal and Life Course Studies* 2019;**10:** 27-50.
31. Azur MJ, Stuart EA, Frangakis C, Leaf PJ. Multiple imputation by chained equations: what is it and how does it work? *Int J Methods Psychiatr Res* 2011;**20:** 40-49.
32. Hope S, Pearce A, Cortina-Borja M, Chittleborough C, Barlow J, Law C. Modelling the potential for parenting skills interventions to reduce inequalities and population prevalence of children's mental health problems: Evidence from the Millennium Cohort Study. *SSM - Population Health* 2021;**14:** 100817.
33. Camacho C, Straatmann VS, Day JC, Taylor-Robinson D. Development of a predictive risk model for school readiness at age 3 years using the UK Millennium Cohort Study. *BMJ Open* 2019;**9:** e024851.
34. Manly CA, Wells RS. Reporting the use of multiple imputation for missing data in higher education research. *Research in Higher Education* 2015;**56:** 397-409.
35. Woods AD, Davis-Kean P, Halvorson M, King K, Logan JR, Xu M*, et al.* Missing Data and Multiple Imputation Decision Tree. *PsyArXiv* 2021.
36. Libuy N, Bann D, Fitzsimons E. Inequalities in body mass index, diet and physical activity in the UK: Longitudinal evidence across childhood and adolescence. *SSM - Population Health* 2021;**16:** 100978.
37. Madigan A, Daly M. Socioeconomic status and depressive symptoms and suicidality: The role of subjective social status. *Journal of Affective Disorders* 2023;**326:** 36-43.

Supplementary tables

Table S1. Associations between indexes of psychological well-being related measures and weight changes to a follow-up of age 17 with additional adjustment for BMI z-score at baseline

Psychological well-being related measures	Baseline: Age 11 (n = 4,556)			Baseline: Age 14 (n = 3,791)		
	Reversal vs. persistence of overweight/obesity by age 17[a]			Reversal vs. persistence of overweight/obesity by age 17[b]		
	**OR**	**95% CI**	**p-value**	**OR**	**95% CI**	**p-value**
Caregiver-reported child mental health	1.12	1.00, 1.27	0.057	1.01	0.88, 1.17	0.858
Child-reported psychosocial well-being	1.27	1.13, 1.43	<0.001*	1.01	0.88, 1.16	0.881

**p-value remained statistically significant after correcting multiple comparisons using Benjamini-Hochberg (BH) adjustment method (see Tables S8 and S9)*

OR = odds ratio; CI = confidence intervals

Indexes of psychological well-being related measures were in z-scores (mean = 0; SD = 1)

[a]Both indexes were fitted in the same regression model, adjusting for sex, ethnicity, family structure, caregiver education, caregiver employment, family income, pubertal status at age 11, changes in pubertal status from age 11 to 14, and baseline BMI-z scores.

[b]Both indexes were fitted in the same regression model, adjusting for sex, ethnicity, family structure, caregiver education, caregiver employment, family income, pubertal status at age 14, and baseline BMI-z scores.

Table S2. Interaction between indexes of psychological well-being related measures and index of socioeconomic status in predicting weight changes to a follow-up of age 17

**Reference:**	**Baseline: Age 11 (n = 4,556)**						**Baseline: Age 14 (n = 3,791)**					
**Psychological well-being related measures * SES**	**Reversal vs. persistence of overweight/obesity by age 17[a]**			**Residualised change scores from age 11 to 17[a]**			**Reversal vs. persistence of overweight/obesity by age 17[b]**			**Residualised change scores from age 14 to 17[b]**		
	**OR**	**95% CI**	**p-value**	**β**	**95% CI**	**p-value**	**OR**	**95% CI**	**p-value**	**β**	**95% CI**	**p-value**
Caregiver-reported child mental health * SES	1.03	0.91, 1.17	0.612	-0.01	-0.05, 0.03	0.711	1.08	0.94, 1.24	0.255	-0.00	-0.05, 0.04	0.835
Child-reported psychosocial well-being * SES	1.00	0.90, 1.11	0.992	0.00	-0.03, 0.04	0.878	1.05	0.93, 1.17	0.432	-0.02	-0.05, 0.02	0.403

OR = odds ratio; β = regression coefficient; CI = confidence intervals; SES = socioeconomic status

Indexes of psychological well-being related measures and socioeconomic status were in z-scores (mean = 0; SD = 1)

[a]Interaction terms between indexes of psychological well-being related measures and an index of socioeconomic status were fitted in the same regression model, adjusting sex, ethnicity, family structure, index of socioeconomic status, pubertal status at age 11, and changes in pubertal status from age 11 to 14.

[b]Interaction terms between indexes of psychological well-being related measures and an index of socioeconomic status were fitted in the same regression model, adjusting sex, ethnicity, family structure, index of socioeconomic status, and pubertal status at age 14.

Table S3. Interaction between indexes of psychological well-being related measures and sex in predicting weight changes to a follow-up of age 17

**Reference:**	**Baseline: Age 11 (n = 4,556)**						**Baseline: Age 14 (n = 3,791)**					
**Psychological well-being related measures * males**	**Reversal vs. persistence of overweight/obesity by age 17[a]**			**Residualised change scores from age 11 to 17[a]**			**Reversal vs. persistence of overweight/obesity by age 17[b]**			**Residualised change scores from age 14 to 17[b]**		
	**OR**	**95% CI**	**p-value**	**β**	**95% CI**	**p-value**	**OR**	**95% CI**	**p-value**	**β**	**95% CI**	**p-value**
Caregiver-reported child mental health * females	1.12	0.87, 1.44	0.381	-0.03	-0.12, 0.05	0.451	0.97	0.73, 1.30	0.847	0.00	-0.08, 0.09	0.930
Child-reported psychosocial well-being * females	0.96	0.77, 1.18	0.672	0.02	-0.06, 0.10	0.556	0.79	0.60, 1.03	0.081	0.03	-0.05, 0.11	0.435

OR = odds ratio; β = regression coefficient; CI = confidence intervals

Indexes of psychological well-being related measures and socioeconomic status were in z-scores (mean = 0; SD = 1)

[a]*Interaction terms between indexes of psychological well-being related measures and sex were fitted in the same regression model, adjusting sex, ethnicity, family structure, caregiver education, caregiver employment, family income, pubertal status at age 11, and changes in pubertal status from age 11 to 14.*

[b]*Interaction terms between indexes of psychological well-being related measures and sex were fitted in the same regression model, adjusting sex, ethnicity, family structure, caregiver education, caregiver employment, family income, and pubertal status at age 14.*

Table S4. Associations between indexes of psychological well-being related measures and weight changes with age 11 as the baseline and age 14 as the follow-up

**Psychological well-being related measures**	**Baseline: Age 11, Follow-up: Age 14 (n = 4,557)**					
	**Reversal vs. persistence of overweight/obesity by age 14**			**Residualised change scores from age 11 to 14**		
	**OR**	**95% CI**	**p-value**	**β**	**95% CI**	**p-value**
Caregiver-reported child mental health	1.07	0.95, 1.21	0.238	-0.04	-0.08, -0.01	0.024
Child-reported psychosocial well-being	1.20	1.07, 1.35	0.003*	-0.04	-0.07, -0.01	0.008*

**p-value remained statistically significant after correcting multiple comparisons using Benjamini-Hochberg (BH) adjustment method (see Tables S8 and S9)*

OR = odds ratio; β = regression coefficient; CI = confidence intervals

Indexes of psychological well-being related measures were in z-scores (mean = 0; SD = 1)

Both indexes were fitted in the same regression model, adjusting for sex, ethnicity, family structure, caregiver education, caregiver employment, family income, pubertal status at age 11, and changes in pubertal status from age 11 to 14.

Table S5. Interactions between baselines and indexes of psychological well-being related measures in predicting weight changes to a follow-up of age 17

**Reference:** **Psychological well-being related measures * Age 14**	**n = 8,347**					
	**Reversal vs. persistence of overweight/obesity by age 17**			**Residualised change scores from ages 11 or 14 to 17**		
	**OR**	**95% CI**	**p-value**	**β**	**95% CI**	**p-value**
Caregiver-reported child mental health * Age 11	1.08	0.91, 1.28	0.399	-0.06	-0.12, 0.00	0.067
Child-reported psychosocial well-being * Age 11	1.24	1.05, 1.47	0.014*	-0.08	-0.13, -0.03	0.003*

**p-value remained statistically significant after correcting multiple comparisons using Benjamini-Hochberg (BH) adjustment method (see Tables S8 and S9)*

OR = odds ratio; β = regression coefficient; CI = confidence intervals

Indexes of psychological well-being related measures were in z-scores (mean = 0; SD = 1)

Interaction terms between baselines and indexes of psychological well-being related measures were fitted in the same regression model, adjusting sex, ethnicity, family structure, caregiver education, caregiver employment, family income, and baseline pubertal status.

Table S6. Associations between individual psychological well-being related measures and weight changes age 11 as the baseline and age 14 as the follow-up

**Psychological well-being related measures**	**Baseline: Age 11, Follow-up: Age 14 (n = 4,557)**					
	**Reversal vs. persistence of overweight/obesity by age 14**			**Residualised change scores from age 11 to 14**		
	**OR**	**95% CI**	**p-value**	**β**	**95% CI**	**p-value**
Internalising symptoms	0.87	0.78, 0.97	0.014*	0.04	0.01, 0.07	0.008*
Externalising symptoms	0.93	0.83, 1.03	0.152	0.06	0.03, 0.08	<0.001*
Self-esteem	1.13	1.04, 1.24	0.006*	-0.02	-0.05, 0.00	0.089
Depressive symptoms	0.96	0.86, 1.07	0.470	0.05	0.02, 0.07	0.001*
Life satisfaction	1.17	1.04, 1.31	0.009*	-0.04	-0.07, -0.01	0.003*
Appearance satisfaction	1.19	1.08, 1.33	0.001*	-0.03	-0.05, 0.00	0.070
Peer bullying	0.93	0.84, 1.03	0.186	0.04	0.01, 0.07	0.003*
Callous/unemotional traits	0.92	0.82, 1.04	0.175	0.03	-0.00, 0.06	0.071

**p-value remained statistically significant after correcting multiple comparisons using Benjamini-Hochberg (BH) adjustment method (see Tables S8 and S9)*

OR = odds ratio; β = regression coefficient; CI = confidence intervals

All individual psychological well-being related measures were in z-scores (mean = 0; SD = 1)

Separate regression models were developed for each individual psychological well-being related measure, adjusting for sex, ethnicity, family structure, caregiver education, caregiver employment, family income, pubertal status at age 11, and changes in pubertal status from age 11 to 14.

Table S7. Interactions between baselines and individual psychological well-being related measures in predicting weight changes to a follow-up of age 17

**Reference:**	n = 8,347					
**Psychological well-being related measure * Age 14**	**Reversal vs. persistence of overweight/obesity by age 17**			**Residualised change scores from ages 11 or 14 to 17**		
	**OR**	**95% CI**	**p-value**	**β**	**95% CI**	**p-value**
Internalising symptoms * Age 11	0.90	0.77, 1.04	0.159	0.07	0.01, 0.12	0.014*
Externalising symptoms * Age 11	0.90	0.77, 1.06	0.202	0.05	0.00, 0.10	0.037
Self-esteem * Age 11	1.14	0.96, 1.34	0.133	-0.06	-0.10, -0.01	0.027
Depressive symptoms * Age 11	0.77	0.67, 0.89	<0.001*	0.09	0.04, 0.14	0.001*
Life satisfaction * Age 11	1.27	1.06, 1.53	0.011*	-0.07	-0.12, -0.02	0.006*
Appearance satisfaction * Age 11	1.12	0.96, 1.31	0.165	-0.06	-0.12, -0.01	0.032
Peer bullying * Age 11	0.96	0.82, 1.13	0.613	0.07	0.02, 0.12	0.011*

**p-value remained statistically significant after correcting multiple comparisons using Benjamini-Hochberg (BH) adjustment method (see Tables S8 and S9)*

OR = odds ratio; β = regression coefficient; CI = confidence intervals

All individual psychological well-being related measures were in z-scores (mean = 0; SD = 1)

Separate regression models were developed for an interaction between baseline and each individual psychological well-being related measure, adjusting for sex, ethnicity, family structure, caregiver education, caregiver employment, family income, and baseline pubertal status.

Only individual psychological well-being related measures consistently available in both Sweeps 5 and 6 were examined (n = 7).

Table S8. Benjamini-Hochberg (BH) adjustment method for the additional analyses of associations between psychological well-being related measures and reversal (vs. persistence)

Reversal vs. persistence				
**Tables**	**Variables - baseline, follow-up**	**p-value**	**Rank**	**(i/n)q**
S1	Child-reported psychosocial well-being - Sweep 5,7	<0.001	1	0.001
4	Depressive symptoms - Sweep 5,7	<0.001	2	0.002
4	Peer bullying - Sweep 5,7	<0.001	3	0.003
S7	Depressive symptoms - Interaction (5&6), 7	<0.001	4	0.004
4	Appearance satisfaction - Sweep 5,7	0.001	5	0.005
S6	Appearance satisfaction - Sweep 5,6	0.001	6	0.006
4	Internalising symptoms - Sweep 5,7	0.002	7	0.007
4	Externalising symptoms - Sweep 5,7	0.002	8	0.008
4	Life satisfaction - Sweep 5,7	0.003	9	0.009
4	Callous/unemotional traits - Sweep 5,7	0.003	10	0.010
S4	Child-reported psychosocial well-being - Sweep 5,6	0.003	11	0.011
S6	Self-esteem - Sweep 5,6	0.006	12	0.013
S6	Life satisfaction - Sweep 5,6	0.009	13	0.014
S7	Life satisfaction - Interaction (5&6), 7	0.011	14	0.015
4	Self-esteem - Sweep 5,7	0.012	15	0.016
S5	Child-reported psychosocial well-being - Interaction (5&6), 7	0.014	16	0.017
S6	Internalising symptoms - Sweep 5,6	**0.014**	**17**	**0.018**
4	Peer bullying - Sweep 6,7	0.031	18	0.019
S1	Caregiver-reported child mental health - Sweeps 5,7	0.057	19	0.020
4	Appearance satisfaction - Sweep 6,7	0.059	20	0.021
S3	Child-reported psychosocial well-being - Sweep 6,7	0.081	21	0.022
S7	Self-esteem - Interaction (5&6), 7	0.133	22	0.023
S6	Externalising symptoms - Sweep 5,6	0.152	23	0.024
S7	Internalising symptoms - Interaction (5&6), 7	0.159	24	0.025
S7	Appearance satisfaction - Interaction (5&6), 7	0.165	25	0.026
4	Internalising symptoms - Sweep 6,7	0.166	26	0.027
S6	Callous/unemotional traits - Sweep 5,6	0.175	27	0.028
S6	Peer bullying - Sweep 5,6	0.186	28	0.029
S7	Externalising symptoms - Interaction (5&6), 7	0.202	29	0.030
S4	Caregiver-reported child mental health - Sweep 5,6	0.238	30	0.031
S2	Caregiver-reported child mental health - Sweep 6,7	0.255	31	0.032
4	Social support - Sweep 6,7	0.328	32	0.033
S3	Caregiver-reported child mental health - Sweep 5,7	0.381	33	0.034
S5	Caregiver-reported child mental health - Interaction (5&6), 7	0.399	34	0.035
4	Externalising symptoms - Sweep 6,7	0.405	35	0.036
S2	Child-reported psychosocial well-being - Sweep 6,7	0.432	36	0.038
S6	Depressive symptoms - Sweep 5,6	0.470	37	0.039
4	Self-esteem - Sweep 6,7	0.502	38	0.040
4	Life satisfaction - Sweep 6,7	0.508	39	0.041
S2	Caregiver-reported child mental health - Sweep 5,7	0.612	40	0.042
S7	Peer bullying - Interaction (5&6), 7	0.613	41	0.043

S3	Child-reported psychosocial well-being - Sweep 5,7	0.672	42	0.044
4	Depressive symptoms - Sweep 6,7	0.808	43	0.045
S3	Caregiver-reported child mental health - Sweep 6,7	0.847	44	0.046
S1	Caregiver-reported child mental health - Sweep 6,7	0.858	45	0.047
S1	Child-reported psychosocial well-being - Sweep 6,7	0.881	46	0.048
4	Online bullying - Sweep 6,7	0.918	47	0.049
S2	Child-reported psychosocial well-being - Sweep 5,7	0.992	48	0.050

i = the individual p-value's rank; n = total number of tests (48); q = the false discovery rate (5%)

The largest p-value lower than the Benjamini-Hochberg critical value is 0.014 (Rank 17). This p-value and all smaller p-values will be considered statistically significant.

Table S9. Benjamini-Hochberg (BH) adjustment method for the additional analyses of associations between psychological well-being related measures and residualised change scores

	Residualised changed scores			
**Tables**	**Variables - baseline, follow-up**	**p-value**	**Rank**	**(i/n)q**
4	Internalising symptoms - Sweep 5,7	<0.001	1	0.001
4	Externalising symptoms - Sweep 5,7	<0.001	2	0.002
4	Depressive symptoms - Sweep 5,7	<0.001	3	0.003
4	Peer bullying - Sweep 5,7	<0.001	4	0.005
S6	Externalising symptoms - Sweep 5,6	<0.001	5	0.006
S6	Depressive symptoms - Sweep 5,6	0.001	6	0.007
S7	Depressive symptoms - Interaction (5&6), 7	0.001	7	0.008
4	Life satisfaction - Sweep 5,7	0.002	8	0.009
S5	Child-reported psychosocial well-being - Interaction (5&6), 7	0.003	9	0.010
S6	Life satisfaction - Sweep 5,6	0.003	10	0.011
S6	Peer bullying - Sweep 5,6	0.003	11	0.013
S7	Life satisfaction - Interaction (5&6), 7	0.006	12	0.014
S4	Child-reported psychosocial well-being - Sweep 5,6	0.008	13	0.015
S6	Internalising symptoms - Sweep 5,6	0.008	14	0.016
S7	Peer bullying - Interaction (5&6), 7	0.011	15	0.017
S7	Internalising symptoms - Interaction (5&6), 7	**0.014**	**16**	**0.018**
S4	Caregiver-reported child mental health - Sweep 5,6	0.024	17	0.019
S7	Self-esteem - Interaction (5&6), 7	0.027	18	0.020
S7	Appearance satisfaction - Interaction (5&6), 7	0.032	19	0.022
S7	Externalising symptoms - Interaction (5&6), 7	0.037	20	0.023
4	Appearance satisfaction - Sweep 5,7	0.064	21	0.024
S5	Caregiver-reported child mental health - Interaction (5&6), 7	0.067	22	0.025
S6	Appearance satisfaction - Sweep 5,6	0.070	23	0.026
S6	Callous/unemotional traits - Sweep 5,6	0.071	24	0.027
4	Peer bullying - Sweep 6,7	0.076	25	0.028
4	Callous/unemotional traits - Sweep 5,7	0.083	26	0.030
S6	Self-esteem - Sweep 5,6	0.089	27	0.031
4	Externalising symptoms - Sweep 6,7	0.091	28	0.032
4	Self-esteem - Sweep 5,7	0.100	29	0.033
4	Depressive symptoms - Sweep 6,7	0.259	30	0.034
4	Online bullying - Sweep 6,7	0.287	31	0.035
4	Social support - Sweep 6,7	0.356	32	0.036
4	Internalising symptoms - Sweep 6,7	0.374	33	0.038
S2	Child-reported psychosocial well-being - Sweep 6,7	0.403	34	0.039
S3	Child-reported psychosocial well-being - Sweep 6,7	0.435	35	0.040
S3	Caregiver-reported child mental health - Sweep 5,7	0.451	36	0.041
S3	Child-reported psychosocial well-being - Sweep 5,7	0.556	37	0.042
4	Self-esteem - Sweep 6,7	0.673	38	0.043
S2	Caregiver-reported child mental health - Sweep 5,7	0.711	39	0.044
4	Life satisfaction - Sweep 6,7	0.714	40	0.045
S2	Caregiver-reported child mental health - Sweep 6,7	0.835	41	0.047

S2	Child-reported psychosocial well-being - Sweep 5,7	0.878	42	0.048
4	Appearance satisfaction - Sweep 6,7	0.928	43	0.049
S3	Caregiver-reported child mental health - Sweep 6,7	0.930	44	0.050

i = the individual p-value's rank; n = total number of tests (44); q = the false discovery rate (5%)

The largest p-value lower than the Benjamini-Hochberg critical value is 0.014 (Rank 16). This p-value and all smaller p-values will be considered statistically significant.

Table S10. Associations between indexes of psychological well-being related measures and weight changes to a follow-up of age 17 **using a case-wise deletion approach**

**Psychological well-being related measures**	**Baseline: Age 11 (n = 2,411)**						**Baseline: Age 14 (n = 2,629)**					
	**Reversal vs. persistence of overweight/obesity by age 17[a]**			**Residualised change scores from age 11 to 17[a]**			**Reversal vs. persistence of overweight/obesity by age 17[b]**			**Residualised change scores from age 14 to 17[b]**		
	**OR**	**95% CI**	**p-value**	**β**	**95% CI**	**p-value**	**OR**	**95% CI**	**p-value**	**β**	**95% CI**	**p-value**
Caregiver-reported child mental health	1.18	1.03, 1.37	0.022	-0.08	-0.13, -0.03	0.001	1.07	0.95, 1.21	0.283	-0.03	-0.07, 0.01	0.085
Child-reported psychosocial well-being	1.18	1.04, 1.34	0.011	-0.07	-0.11, -0.02	0.003	1.05	0.92, 1.19	0.469	-0.01	-0.05, 0.03	0.670

OR = odds ratio; β = regression coefficient; CI = confidence intervals

Indexes of psychological well-being related measures were in z-scores (mean = 0; SD = 1)

[a]*Both indexes were fitted in the same regression model, adjusting for sex, ethnicity, family structure, caregiver education, caregiver employment, family income, pubertal status at age 11, and changes in pubertal status from age 11 to 14.*

[b]*Both indexes were fitted in the same regression model, adjusting for sex, ethnicity, family structure, caregiver education, caregiver employment, family income, and pubertal status at age 14.*
